# m6A Methylation Analysis of Differentially Expressed Genes in Skin Tissues of Coarse and Fine Type Liaoning Cashmere Goats

**DOI:** 10.3389/fgene.2019.01318

**Published:** 2020-01-22

**Authors:** Yanru Wang, Yuanyuan Zheng, Dan Guo, Xinghui Zhang, Suling Guo, Taiyu Hui, Chang Yue, Jiaming Sun, Suping Guo, Zhixian Bai, Weidong Cai, Xinjiang Zhang, Yixing Fan, Zeying Wang, Wenlin Bai

**Affiliations:** ^1^ College of Animal Science & Veterinary Medicine, Shenyang Agricultural University, Shenyang, China; ^2^ Academy of Animal Husbandry Science of Liaoning Province, Liaoyang, China; ^3^ Prosperous Community, Huade, China

**Keywords:** MeRIP-seq, RNA-seq, m6A modification, cashmere fineness, m6A-modified genes

## Abstract

N6-methyladenosine (m6A) is the most common internal modification in mRNAs of all higher eukaryotes. Here we perform two high-throughput sequencing methods, m6A-modified RNA immunoprecipitation sequence (MeRIP-seq) and RNA sequence (RNA-seq) to identify key genes with m6A modification in cashmere fiber growth. A total of 9,085 m6A sites were differentially RNA m6A methylated as reported from by MeRIP-seq, including 7,170 upregulated and 1,915 downregulated. In addition, by comparing m6A-modified genes between the fine-type Liaoning cashmere goat (FT-LCG) and coarse-type Liaoning Cashmere Goat (CT-LCG) skin samples, we obtain 1,170 differentially expressed genes. In order to identify the differently methylated genes related to cashmere fiber growth, 19 genes were selected to validate by performing qRT-PCR in FT-LCG and CT-LCG. In addition, GO enrichment analysis shows that differently methylated genes are mainly involved in keratin filament and intermediate filament. These findings provide a theoretical basis for future research on the function of m6A modification during the growth of cashmere fiber.

## Introduction

In the 1970s, scientists discovered m6A modification, which can occur on RNA adenine (A) such as mRNA and long non-coding RNA (Dubin and Taylor, 1975). Subsequent studies have shown that methylation of m6A exists in the mRNA of different prokaryotes, eukaryotes, and viruses (Desrosiers et al., 1974; Dubin and Taylor, 1975; Furuichi and Miura, 1975; Beemon and Keith, 1977). However, the clear function and mechanism of the m6A modification were largely unclear until recently. Until now, more than 150 types of posttranscriptional modifications have been identified in RNA among all living organisms (Boccaletto et al., 2018). m6A is a chemical modification that exists in a variety of RNA and has the highest abundance in mRNA (Yang et al., 2018).

Earlier studies on m6A mainly focused on the methylation modification at the mRNA 5'Cap, including the maintenance of mRNA stability, mRNA precursor shear, polyadenylation, mRNA transport, and translation initiation. The modification of 3' polyA contributes to nuclear transport, initiation of translation, and maintenance of structural stability of mRNA with polyA binding protein. In recent years, there have been more and more studies on internal modification of RNA, including N6 methyladenosine modification (m6A), N1 methyladenosine modification (m1A), methylcytosine modification (m5C), and pseudouridine modification (Ψ) (Weng et al., 2018). m6A is present transcriptome-wide in over 25% of all RNAs (Hibar et al., 2015), as the most common methylation modification in RNA, m6A is mainly concentrated in long exons, stop codons, and in 3′UTRs (Dominissini et al., 2012). As similar with DNA and histone methylation, m6A RNA methylation is also dynamic and reversible in mammalian cells and it has been proposed as another form of epigenetic regulation. In mammals, m6A modification occurs by a methyltransferase complex (writers) mainly consisting of METTL3, METTL14, WTAP, and other components. The demethylases (erasers) FTO and ALKBH5 can mediate the demethylation of m6A modification (Li et al., 2015; Meyer and Jaffrey, 2017).

A large amount of original work of m6A is still concentrated in human, mouse, and other model animals. Studies have shown that m6A methylation is associated with obesity (Ben-Haim et al., 2015). Lin indicated that m6A was dynamically regulated and played crucial roles to shape gene expression in spermatogonial stem cells development and during murine spermatogenesis (Lin et al., 2017). In Drosophila melanogaster, the m6A-deficient Drosophila melanogaster could survive, but could not fly and its fertility declined (Haussmann et al., 2016). Recent studies have demonstrated that the deletion of YTHDF2 resulted in the non-regulation of transcripts of related genes during oocyte maturation, leading to the infertility of specific YTHDF2 deficient female mice (Ivanova et al., 2017). Wang indicated that both m6A in total RNA and expression of METTL3 were significantly decreased in mouse hippocampus after traumatic brain injury (Wang et al., 2019). But there is no relevant reference on cashmere goats.

Cashmere goat is a kind of goat breed which mainly produces cashmere. It produces more cashmere and has better cashmere quality. Cashmere produced by the secondary hair follicles of cashmere goat is a shiny and comfortable natural fiber (Xiaolong et al., 2016; Bai et al., 2018). The fineness of cashmere is the most important factor affecting cashmere production. It is an important index to evaluate the quality of cashmere in many countries. The standard of cashmere classification in China is that fineness between 14.5 to 16.0 μm is fine type, between 16.0 to18.5 μm is coarse type (GB18267-2013). The fineness of cashmere has been determined as early as the development of hair follicles, the development of hair follicles has a certain impact on the fineness of cashmere. Therefore, in order to improve the quality of cashmere, the fineness of cashmere has become more and more popular (Bai et al., 2016; Zhang et al., 2018). Investigations indicated that many genes might widely participate in the growth regulation of cashmere fiber (Stenn and Paus, 2001; Liu et al., 2016). Many studies have identified some genes associated with the growth and properties of wool fibers in sheep and goats, such as DSG1, IGF-IR, KRTAPs, ILK, as well as the KRT and KRTAP genes (Rufaut et al., 1999; Bin et al., 2006; Yu et al., 2011; Yang et al., 2012; Liu et al., 2015). In the past few decades, the regulators of cashmere fiber growth have been studied at several levels, including genes with methylation characteristics (Bai et al., 2013; Fan et al., 2015; Bai et al., 2017). Given the indispensable function of RNA m6A modification in regulating gene express and involving in various bioprocesses, it is reasonable to speculate that regulation of m6A modification might also be associated with the cashmere fineness. Herein, we aim to find the m6A methylation modification landscape of differential genes in the skin of FT-LCG and CT-LCG. And some potential functions of RNA m6A methylation genes in cashmere growth will be explored in the future.

## Materials and Methods

### Sample Collection and Ethics Statement

We collected scapular skin samples from two groups Liaoning Cashmere Goats from Academy of Animal Husbandry Science of Liaoning Province. The two groups goats are three coarse type and three fine type adult female cashmere goats, which had the same feed conditions and growth environment. They are all 1.5 years old. The upper 1/3 of the right scapula along the mid-dorsal and mid-abdominal lines were taken from the lateral skin about 1 cm^2^, and immediately stored in liquid nitrogen until RNA isolation. In addition, three coarse and three fine type skin samples of Liaoning Cashmere Goat were collected for qRT-PCR. All experimental procedures used in this study were approved and conducted according to the guidelines by the Laboratory Animal Management Committee of Shenyang Agricultural University.

### Experimental Procedure

Total RNA was extracted using Trizol reagent (Invitrogen, CA, USA) following the manufacturer's procedure. The total RNA quality and quantity were analysis of Bioanalyzer 2100 and RNA 6000 Nano LabChip Kit (Agilent, CA, USA) with RIN number >7.0. Approximately more than 200ug of total RNA was subjected to isolate Poly (A) mRNA with poly-T oligo attached magnetic beads (Invitrogen). Following purification, the poly(A) mRNA fractions is fragmented into 100-nt-long oligonucleotides using divalent cations under elevated temperature. Then the cleaved RNA fragments were subjected to incubated for 2h at 4°C; with m6A-specific antibody (No. 202003, Synaptic Systems, Germany) in IP buffer (50 mM Tris-HCl, 750 mM NaCl, and 0.5% Igepal CA-630) supplemented with BSA (0.5 μg/ μl). The mixture was then incubated with protein-A beads and eluted with elution buffer (1 × IP buffer and 6.7mM m6A). Eluted RNA was precipitated by 75% ethanol. Eluted m6A-containing fragments (IP) and untreated input control fragments are converted to final cDNA library in accordance with a strand-specific library preparation by dUTP method. The average insert size for the paired-end libraries was 100 ± 50 bp. And then we performed the paired-end 2 × 150 bp sequencing on an Illumina Novaseq™ 6000 platform at the LC-BIO Bio-tech ltd (Hangzhou, China) following the vendor's recommended protocol.

### Bioinformatics Analysis Process

Firstly, Cutadapt and perl scripts in house were used to remove the reads that contained adaptor contamination, low quality bases, and undetermined bases (Martin, 2011). Then sequence quality was verified using fastp. We used HISAT2 to map reads to the genome of Capra_hircus_goat (Version: Capra_hircus_goat_NCBI) with default parameters (Kim et al., 2015). Mapped reads of IP and input libraries were provided for R package exomePeak, which identifies m6A peaks with bed or bam format that can be adapted for visualization on the UCSC genome browser or IGV software (http://www.igv.org/) (Meng et al., 2014). MEME and HOMER were used for *de novo* and known motif finding followed by localization of the motif with respect to peak summit by perl scripts in house (Bailey et al., 2009; Heinz et al., 2010). Called peaks were annotated by intersection with gene architecture using ChIPseeker (Yu et al., 2015). Then StringTie was used to perform expression level for all mRNAs from input libraries by calculating FPKM (FPKM = [total_exon_fragments/mapped_reads (millions) × exon_length (kB)]) (Pertea et al., 2015). The differentially expressed mRNAs were selected with log2 (fold change) > 1 or log2 (fold change) <−1 and *P* < 0.05 by R package edgeR (Robinson et al., 2010).

### Quantitative Real-Time PCR Validation

We detected 19 differently m6A methylated genes for qRT-PCR. In order to validate the differentially methylated genes, total RNAs were synthesized directly to cDNA synthesis by an RT-PCR kit (Takara, Dalian, China). According to the manufacturer's instructions, Real-time PCR was performed using SYBR Green (TaKaRa Biotech, Dalian). The glyceraldehyde-3-phosphate dehydrogenase (GAPDH) gene was used as an internal control to normalize the expression level of genes. Three independent experiments were carried out on three CT-LCG and three FT-LCG skin samples. Nineteen pairs primers were designed by Primer 5 software and listed in the **Supplementary Material**, and all primers were spanning the distal ends of genes. The relative expression levels of differentially expressed genes were analyzed by the 2^−ΔΔCt^ method in qPCR data. The data were indicated as the means ± SE (n = 3). All statistical analyses in the two groups were calculated using a t-test in SPSS statistical software (Version 22.0, Chicago, IL, USA), the difference was significant at *P* < 0.05.

## Results and Analysis

### Sequencing Sequence Statistics and Quality Control

The raw data from IP and input RNA-seq were firstly trimmed by Cutadapt and perl scripts in house to remove the adaptor and low quality data, and the clean reads were obtained. In the MeRIP-seq library, two groups of skin samples were obtained 65964582 and 68530268 raw data reads, 56434060 and 60393976 valid data reads, and the effective reads accounted for 72.94% and 66.49% respectively. In the RNA-seq library, three groups of skin samples were obtained 65680810 and 75067398 raw data reads, 57014334 and 64832184 valid data reads, and the effective reads accounted for 71.84% and 62.20% respectively. The results are shown in **Table 1**. In **Table 1**, Q20% represents proportion of bases with mass value ≥ 20 (sequencing error rate less than 0.01) and Q30% represents proportion of bases with quality value ≥ 30 (sequencing error rate less than 0.001).

**Table 1:** Summary of reads quality control.

Sample	Raw_Reads	Valid_Reads	Valid%	Q20%	Q30%	GC%
FT_LCG_IP	65964582	56434060	72.94	97.96	94.67	54.54
CT_LCG_IP	68530268	60393976	66.49	96.96	92.27	54.38
FT_LCG_input	65680810	57014334	71.84	98.23	95.35	56.29
CT_LCG_input	75067398	64832184	62.20	97.20	92.94	55.53

### Mapping Reads to the Reference Genome

**Figure 1:**
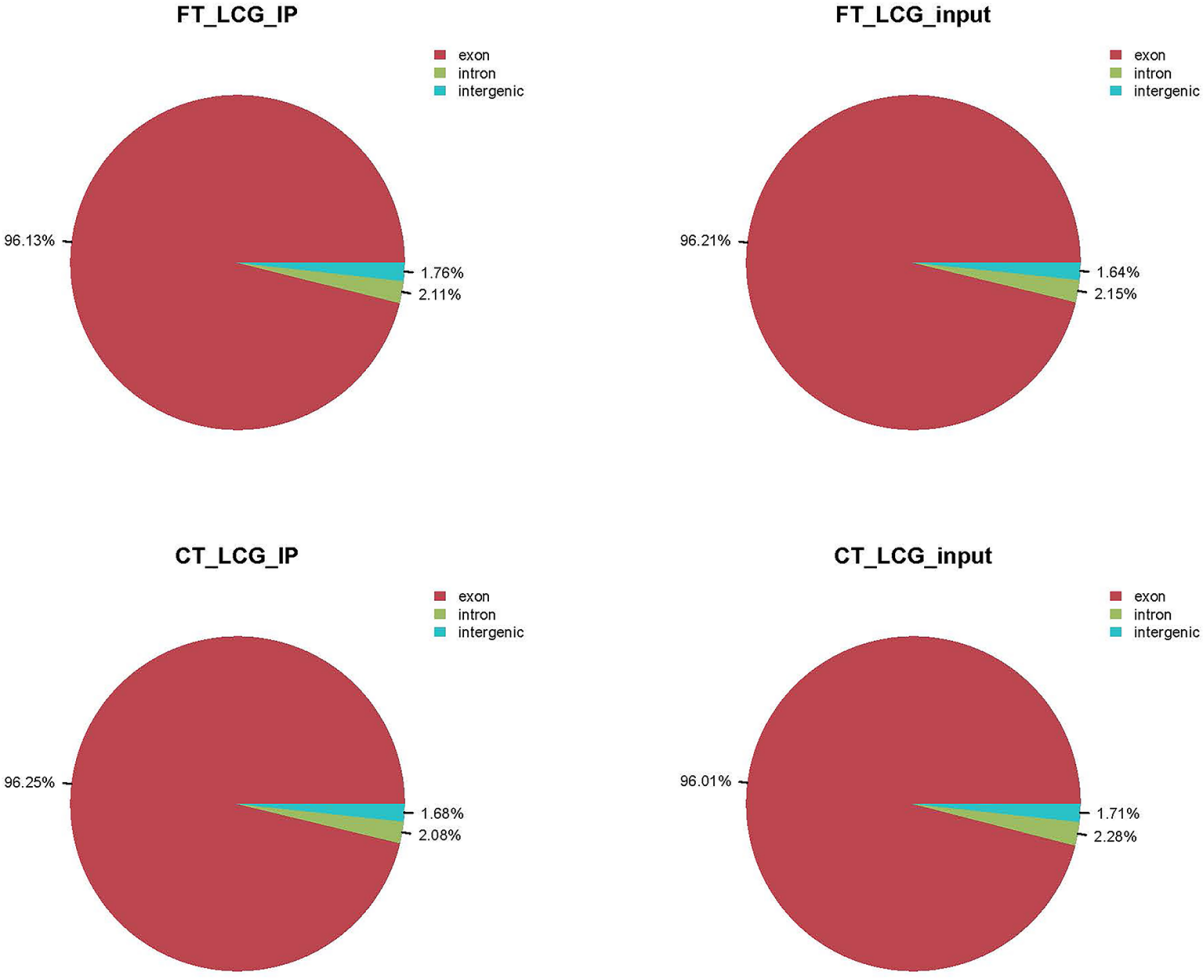
Refer to the genome to compare the regional distribution.

We used HISAT2 to map reads to the genome of *Capra hircus* goat (Version: Capra_hircus_goat_NCBI) with default parameters. By comparing reads with reference sequences, we can make detailed statistics of the alignment of sequencing data. In the m6A-seq library, the mapping ratio of valid data in cashmere goat skin IP samples FT-LCG and CT-LCG were 90.93% and 92.65%. In the RNA-seq library, the mapping ratio of valid reads in cashmere goat skin IP sample FT-LCG and CT-LCG were 90.48% and 92.14%. The ratio of unique mapped reads and the multi-mapped reads were shown in **Table 2**. According to the regional information of the reference genome, it can be defined as the alignment to exon, intron, and intergenic. Under normal circumstances, the percentage of sequencing sequence localization in the Exon region should be the highest. The results of this experiment showed that the ratio of IP samples and Input samples to exons of fine cashmere goat skin was 96.13% and 96.21%. The ratio of IP samples to exons of coarse cashmere goat skin was 96.25%, and the ratio of input samples was 96.01%. The results are shown in **Figure 1**.

**Table 2:** Summary of reads mapping to the goat reference genome.

Sample	Valid reads	Mapped reads	Unique mapped reads	Multi mapped reads
FT_LCG_IP	54178082	49265911 (90.93%)	41202427 (76.05%)	8063484 (14.88%)
CT_LCG_IP	57795130	53545919 (92.65%)	43603327 (75.44%)	9942592 (17.20%)
FT_LCG_input	55762428	50456594 (90.48%)	42786234 (76.73%)	7670360 (13.76%)
CT_LCG_input	63021978	58066249 (92.14%)	48369203 (76.75%)	9697046 (15.39%)

### Identification of m6A Modification Sites and Differential Methylation Analysis

**Figure 2:**
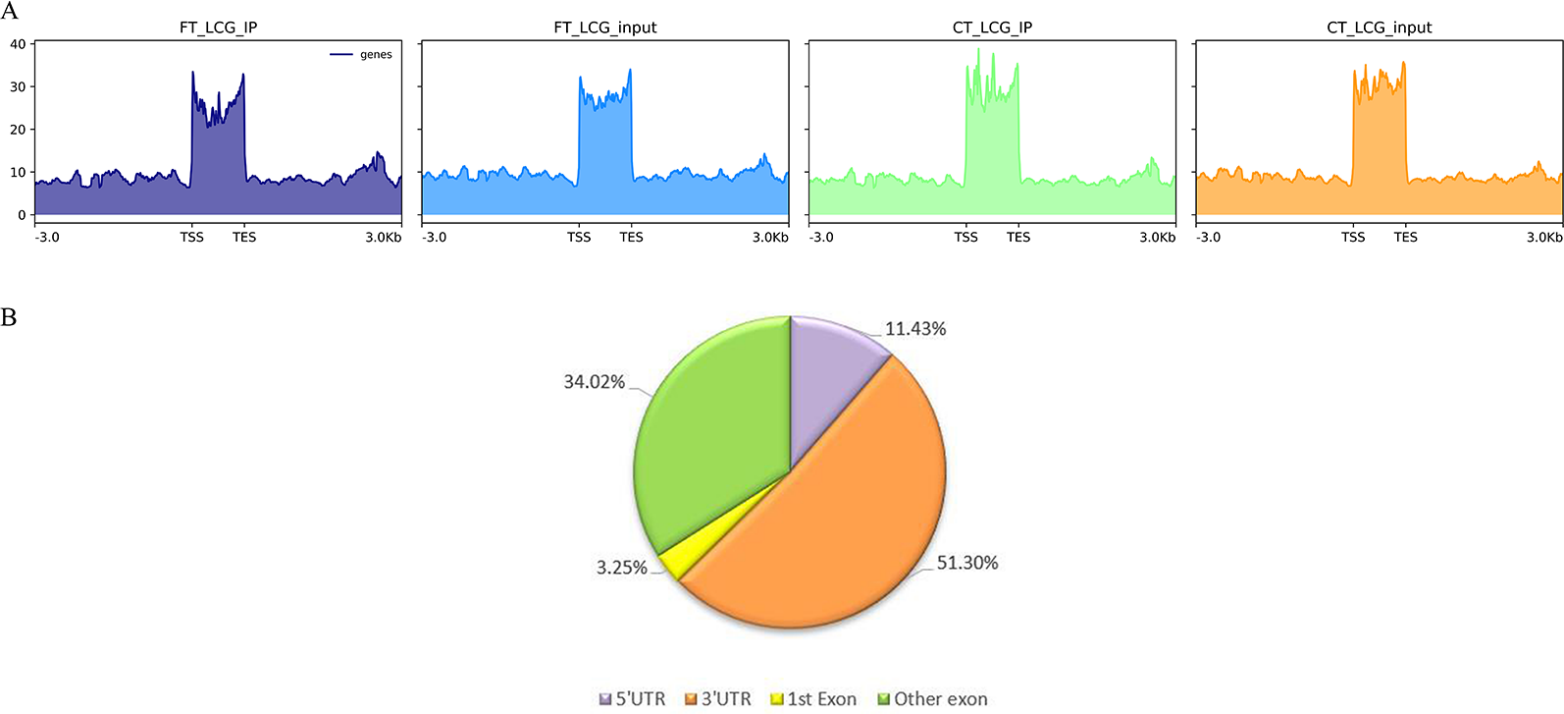
**(A)** The enrichment of reads near TSS at the transcriptome initiation site of the gene. **(B)** The distribution of differential peaks on gene functional elements.

m6A modification sites were identified by using R package exomePeak, which performed peaks scanning in the whole gene range. Peak identification was based on MeRIP-seq and RNA-seq (Input) sequencing data. If *p-value* is less than 0.05, the regions less than the *p-value* are considered to be a peak. We combined all samples to demonstrate the enrichment of reads near TSS at the transcriptome initiation site of the gene. Peak distributions in the combined regions around TSS are shown in heatmap form (**Figure 2A**). Next, we use the ChIPseeker to annotate the difference peaks. At present, the default *P* < 0.05 is the screening threshold of differential peak. A total of 9,085 differential peaks were detected, of which 7,170 m6A peaks were up-regulated and 1,915 peaks were down-regulated. To analyze the distribution of m6A peaks in different regions of the transcript, we divided the transcript into four areas 5′ untranslated region (5'UTR), 3′UTR, 1st exon, and other exon (**Figure 2B**). The differential m6A peaks were mainly enriched in the 3′UTRs. And the top 20 differential m6A peaks were showed in the **Table 3**. Fold change <1 represents hypo-methylation and fold change ≥1 represents hyper-methylation in **Table 3**.

**Table 3:** The top 20 differently expressed m6A peaks.

Gene name	Fold change	Regulation	Chromosome	Peak region	Peak star	Peak end	*p*-value
LOC102181394	100.4267645	Hyper-methylation	10	Exon	66300964	66301204	0.00000000093
KRT4	43.41	Hyper-methylation	5	Exon	26851689	26852283	2.5E-19
C16H1orf159	41.93	Hyper-methylation	16	3′ UTR	49899342	49899402	0.0056
LOC102175116	32.90	Hyper-methylation	18	3′ UTR	61253406	61253915	0.0000000000000005
LOC102185235	31.34	Hyper-methylation	3	3′ UTR	16525	16734	1.3E-28
AGGF1	28.05	Hyper-methylation	10	3′ UTR	92994460	92994580	0.0018
SENP8	23.92	Hyper-methylation	10	3′ UTR	82316906	82317775	5E-64
LOC102188675	23.92	Hyper-methylation	9	3′ UTR	2057892	2059710	0.000000000000005
PABPN1	22.47	Hyper-methylation	10	3′ UTR	79853156	79853276	0.0000058
MED13L	20.97	Hyper-methylation	17	3′ UTR	12307015	12307165	0.000063
EEF2	0.97	Hypo-methylation	7	3′ UTR	90521673	90522091	0
RPL4	0.96	Hypo-methylation	10	3′ UTR	87913288	87914418	0
CEBPD	0.96	Hypo-methylation	14	3′ UTR	62920843	62921673	0
LOC102178315	0.96	Hypo-methylation	23	Exon	22452943	22453927	0
LOC102170546	0.95	Hypo-methylation	19	3′ UTR	40835700	40835905	6.3E-76
RPL23	0.95	Hypo-methylation	19	3′ UTR	39092676	39095474	0
RPL36A	0.95	Hypo-methylation	X	3′ UTR	40653056	40656312	0
LOC102170264	0.94	Hypo-methylation	19	3′ UTR	40842590	40842829	1.3E-64
DNAJB1	0.94	Hypo-methylation	7	3′ UTR	96664725	96665141	0
KRT27	0.94	Hypo-methylation	19	Exon	40625699	40626066	2.5E-34

### Motif Analysis

**Figure 3:**
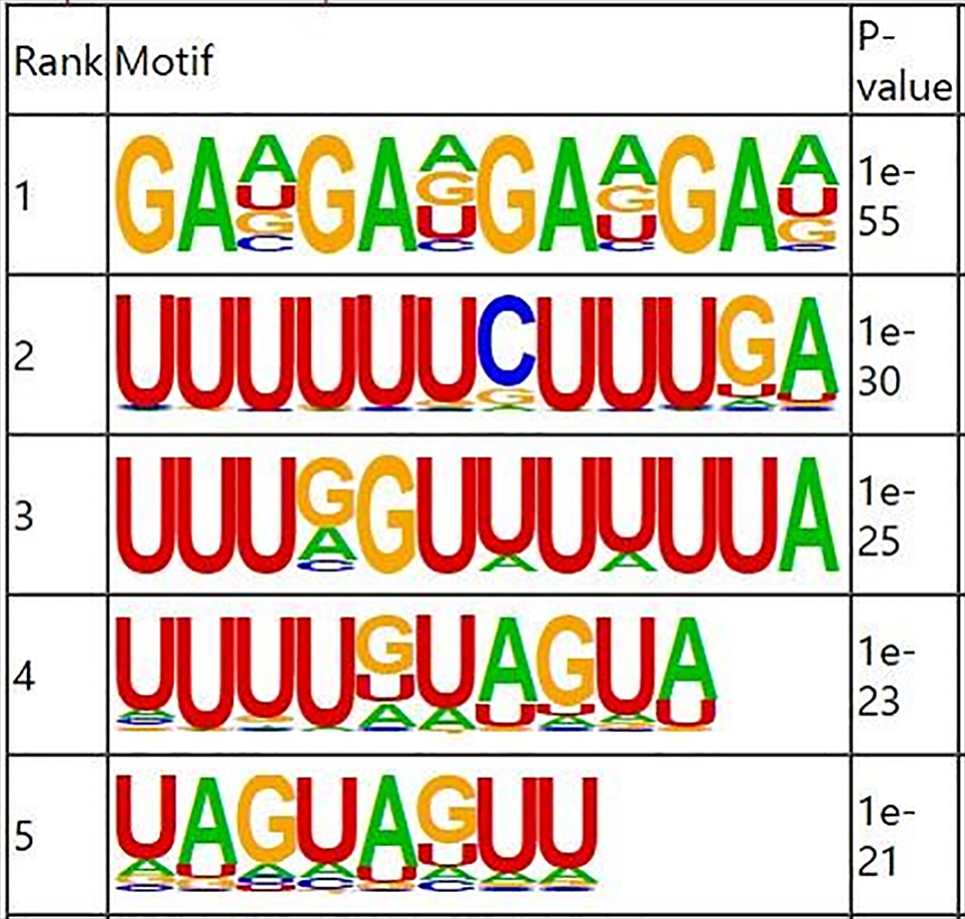
Sequence logo showing the top motifs enriched across differential m6A peaks identified from skin samples.

RNA methylation and demethylation begin with the motif binding of various binding proteins to methylation sites. A motif is essentially a sequence pattern of nucleic acids with biological significance, and these RNA methylation related enzymes recognize and bind to these motifs, thereby affecting gene expression. Motif analysis software MEME was used to search for motifs with high credibility in the peak region, and the width, *e-value, p-value* of each motif, and its general position information in each peak sequence were obtained. We conducted motif prediction for samples and differential results. We sorted the predicted motif in the **Figure 3**. According to the *p-value*, and the smaller the *p-value*, the higher the rank. The most commonly reported RNA motif structures are RRACH (where R = A or G, H = A, C or U). The differential m6A peaks were characterized by the GAAGA motif, which is the first rank motif structures.

### Overall Gene Outcome Analysis and Differential Genes Analysis

**Figure 4:**
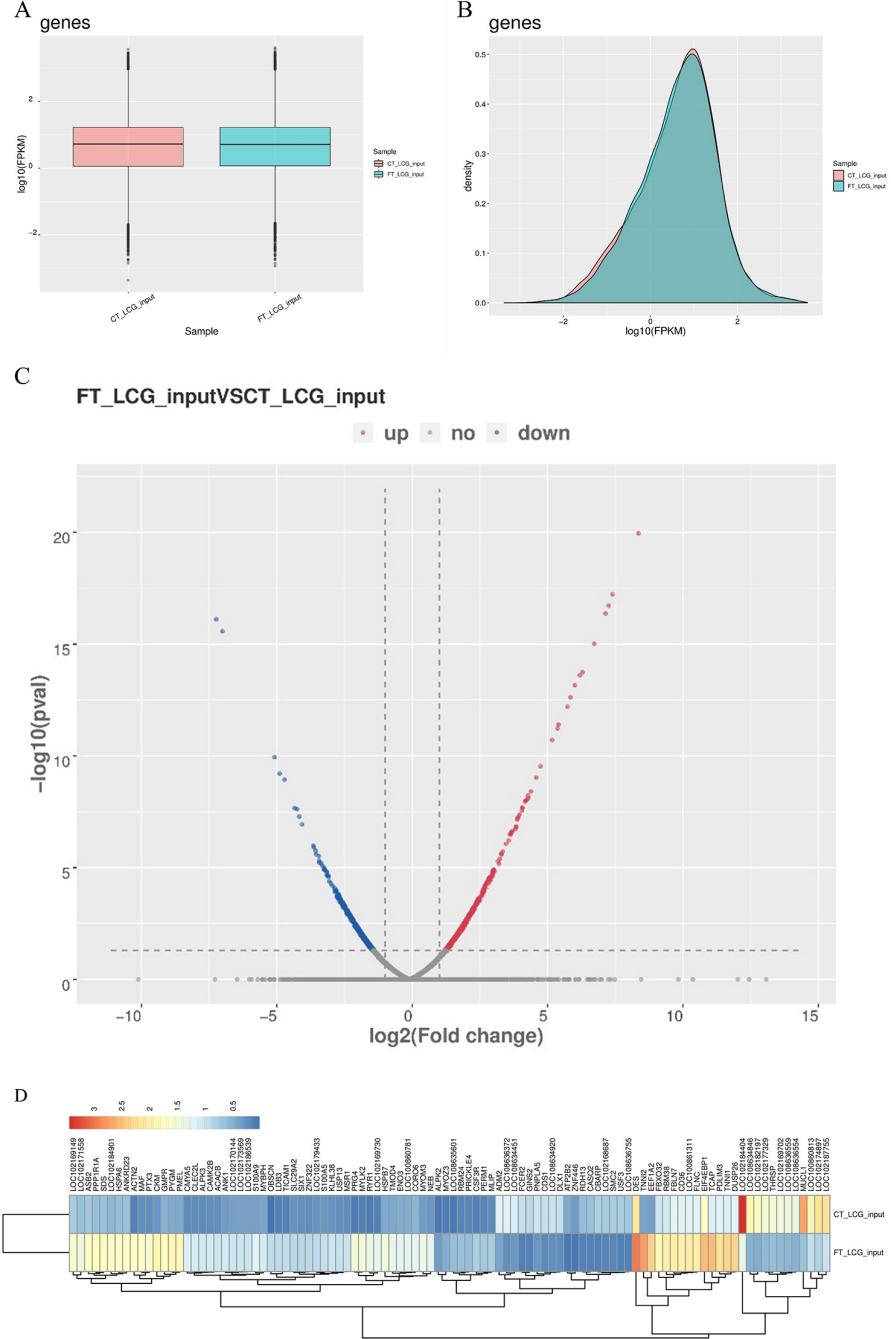
Overview of differentially expressed genes in goats skin samples. **(A)** Gene expression box plot. **(B)** Gene expression density map. **(C)** Volcanic map of differentially expressed genes. [log2(fold change) was used as the horizontal coordinate, and -log10(*p*-value) was used as the vertical coordinate. Volcanic map was drawn for all genes in the differentially expressed analysis. The abscissa represents the multiple change of gene expression in different samples. The ordinate represents the statistical significance of differences in gene expression level. Red represents significantly differentially expressed genes up-regulated, blue represents significantly differentially expressed genes down-regulated, and gray dots represent non-significantly differentially expressed genes.] **(D)** Heatmap of the genes of FT-LCG and CT-LCG.

The level of gene expression is mainly measured by RPKM (reads per kilobase of exon model per million mapped reads) or FPKM (fragments per kilobase of exon model per million mapped reads). In this project, FPKM was used to calculate the expression abundance of known genes in different samples. FPKM represents the number of sequencing fragments contained in the sequence bases of every 1,000 transcripts per million sequencing bases. In short, the FPKM value can be understood as the amount of gene expression. Of the 18,619 genes we have identified, 1,170 were significantly different, while 17,449 were not. Among them, 527 differentially expressed genes were up-regulated and 643 down-regulated. The top 20 differently expressed genes are listed in **Table 4**. Gene expression box plot and gene expression density map are showed in **Figures 4A**, **B**. The overall distribution of differentially expressed genes can be understood by mapping volcanoes in **Figure 4C**. And the heatmap of the genes between FT-LCG and CT-LCG is showed in **Figure 4D**.

**Table 4:** The top 20 differentially expressed genes.

Gene name	Fold change	Regulation	Chromosome	p-value	FT_LCG_input	CT_LCG1_input
TNNI2	268.793905	Up	29	8.91640E-20	463.28	1.72
EEF1A2	199.939716	Up	13	1.19645E-18	256.36	1.28
OBSCN	195.029884	Up	7	2.23394E-18	7.86	0.04
ACTN2	150.516310	Up	28	2.16045E-17	59.17	0.39
PRG4	112.447415	Up	16	5.99177E-16	28.03	0.25
RYR1	75.2314623	Up	18	1.60085E-14	20.47	0.27
MYLK2	74.5979363	Up	13	2.90124E-14	28.83	0.39
CKM	70.6341520	Up	18	3.57699E-14	83.38	1.18
PTX3	51.1155418	Up	1	9.40226E-13	39.77	0.78
PYGM	43.2087950	Up	29	3.24296E-12	77.65	1.80
ADM2	0.11825253	Down	5	0.000024893	0.12	0.02
SMC2	0.11516680	Down	8	0.000022203	0.53	4.58
RDH13	0.11139211	Down	18	0.000020772	0.37	3.36
C18H19orf68	0.10837616	Down	18	0.000016339	0.52	4.83
ATP2B2	0.10696679	Down	22	0.000013947	0.19	1.75
RNF146	0.10608114	Down	9	0.000010708	2.32	21.91
THRSP	0.10601556	Down	29	0.000010374	3.26	30.72
DLEC1	0.10426514	Down	22	0.000014536	0.18	1.69
NPTX2	0.10353937	Down	25	8.81440E-06	1.58	15.30
HELLS	0.10118161	Down	26	0.000011655	0.31	3.11
PNPLA5	0.09786050	Down	5	6.89617E-06	0.97	9.88

### Correlation Analysis Results of Differential Genes and Differential Peaks

In transcriptome sequencing, the results were up-regulated and down-regulated. In the same MeRIP-seq results, the up-regulated methylation of the gene itself can also be found according to the peak abundance changes. Here we conduct a correlation analysis of the contents of the two omics, comparing the transcriptional level with the methylation level. Based on these results, we screened out 19 candidate genes from 256 differentially differently methylated genes associated with the growth of the cashmere, which are listed in **Table 5**. The m6A regulation of these genes are upregulated, the gene regulation are downregulated, and the differences between the two samples are significant.

**Table 5:** Candidate m6A modified genes related to cashmere fineness.

Gene name	Gene ID	M6A regulation	Gene regulation	FPKM.FT-LCG_input	FPKM.CT-LCG_input
LOC108636561	108636561	Up	Down	3.41	24.13
LOC102176522	102176522	Up	Down	32.84	98.10
LOC102184693	102184693	Up	Down	41.07	189.40
LOC108638295	108638295	Up	Down	7.27	23.95
LOC106503204	106503204	Up	Down	35.15	118.97
LOC102173780	102173780	Up	Down	51.92	159.03
LOC108634870	108634870	Up	Down	1.63	6.67
LOC102184223	102184223	Up	Down	296.30	1083.83
KRT79	102182102	Up	Down	84.93	278.57
KRT82	102183763	Up	Down	24.10	101.79
KRT32	102175613	Up	Down	26.46	92.53
KRT26	100860868	Up	Down	18.32	65.63
GJA1	102191242	Up	Down	74.96	277.68
GJB6	102171255	Up	Down	15.17	57.96
VANGL2	102182554	Up	Down	2.35	11.45
ZDHHC21	102189949	Up	Down	1.00	2.67
FZD6	102170617	Up	Down	2.97	10.24
FRS2	102170949	Up	Down	0.55	1.92
EGR3	106501825	Up	Down	9.05	26.07

### GO Analysis and KEGG Pathway Analysis of Differently Methylated Genes

**Figure 5:**
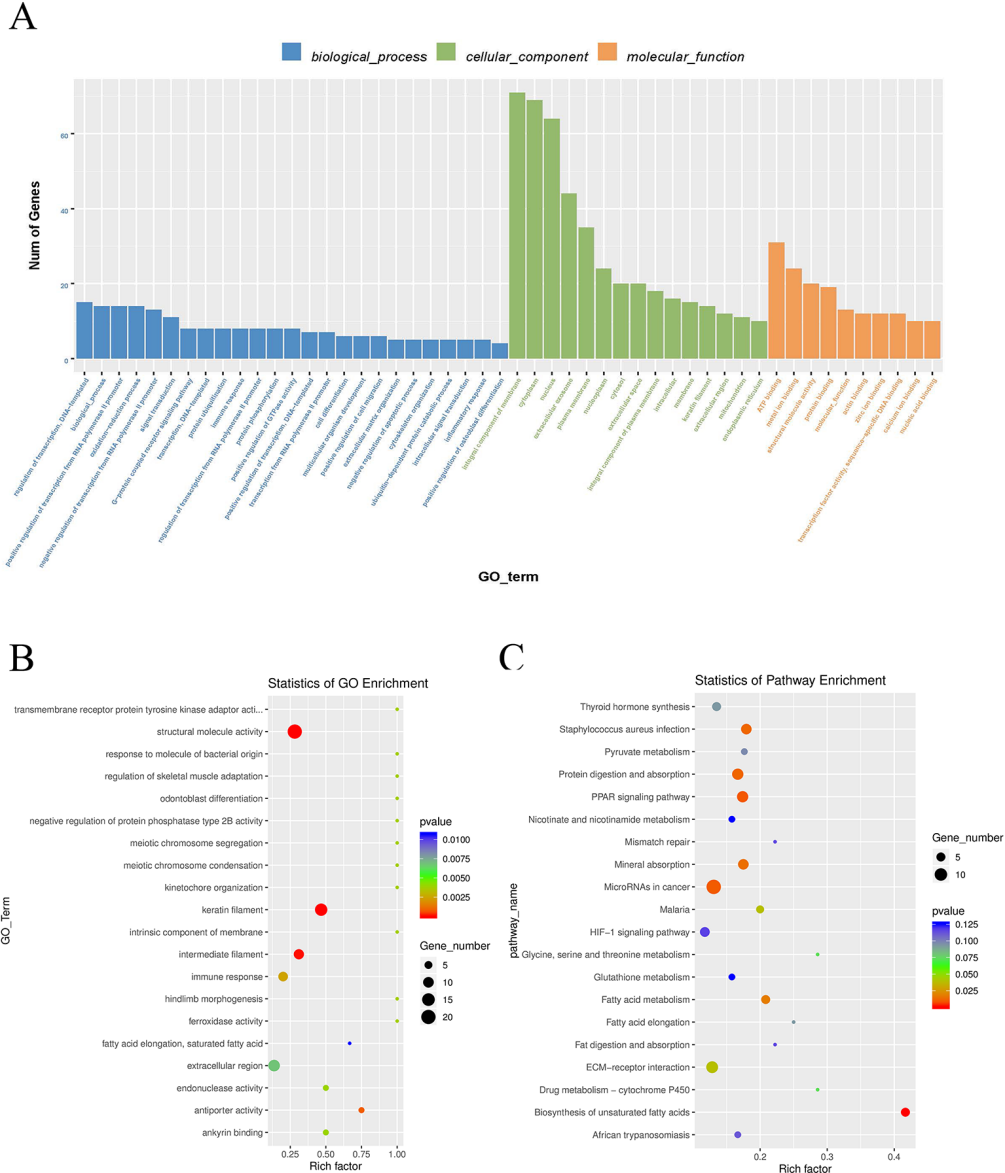
Gene ontology enrichment and KEGG pathway analysis of differential m6A peaks. **(A)** Major enrichment and meaningful GO terms of m6A peaks. **(B)** The top 20 significantly GO enrichment terms. **(C)** The top 20 enriched pathways of m6A peaks.

To explore the physiological and pathological significance of m6A modification, GO (http://www.geneontology.org/) and KEGG (http://www.kegg.jp/) pathway (*P* < 0.05) analysis were performed for differentially peaks. These peaks were enriched for 1,683 GO terms and 225 KEGG pathways. Top 25 in biological process, top 15 in cellular component and top 10 in molecular function are listed in **Figure 5A**. In the **Figure 5B**, Go analysis revealed that the differently methylated genes were significantly enriched in structural molecule activity, keratin filament, and intermediate filament. KEGG Pathway analysis showed that differently methylated genes were significantly associated with microRNA in cancer, PPAR signaling pathway, and protein digestion and absorption (**Figure 5C**).

### Validation of Differentially Expressed Genes by qPCR

**Figure 6:**
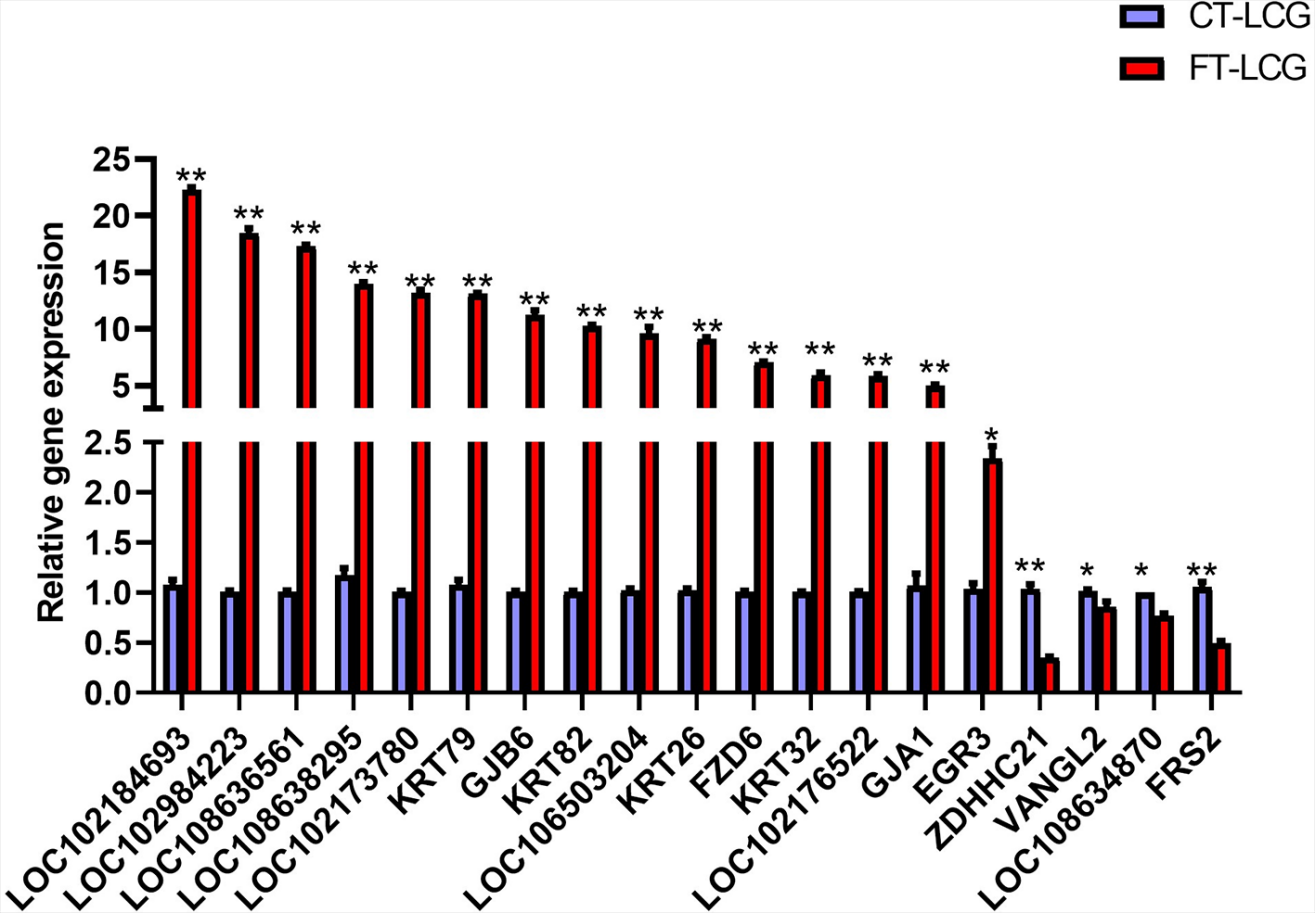
qPCR results of the 19 differentially m6A modified genes in FT-LCG and CT-LCG. The blue-purple column represents CT-LCG, the red column represents FT-LCG, ** represents *P* < 0.01, * represents *P* < 0.05.

To explore the potential role of m6A-modified genes and identify which genes are important to regulate cashmere fiber growth in LCG skin tissue, qRT-PCR was performed. RNA-seq results showed that the expression of differentially expressed genes in FT-LCG skin tissue was higher than that in CT-LCG. qRT-PCR confirmed that these m6A genes did exist in cashmere goat skin tissue. Except ZDHHC21, VANGL2, LOC108634870, FRS2, the variation trend of these genes was consistent with the results of RNA-seq. The results are showed in **Figure 6**.

## Discussion

The same as DNA modifications, a series of reversible modification is present on RNA (Dominissini et al., 2012). Many m6A-related studies have been performed in many mammals, plant, and yeast, but no report has been published on m6A profiling in cashmere goats. Wang et al. identified that 922 m6A peaks, with 370 upregulated and 552 down regulated after traumatic brain injury (Wang et al., 2019). To our knowledge, this is the first comprehensive high-throughput study of RNA methylation in cashmere goat skin tissue. Our data showed that during the growth of cashmere, there are a large number of m6A methylation modification of genes in skin tissues. Further analysis showed that m6A modification may play an important role in cashmere growth by regulating gene expression level.

Herein, two sequencing libraries, namely m6A-seq library (IP) and RNA-seq library (Input), were constructed for high-throughput sequencing, and the results were analyzed with bioinformatics. Using MeRIP-seq method, we detected a large number of m6A methylation peaks in the transcriptome of cashmere goat skin tissue. In this study, 9,085 different peaks were detected, 7,170 m6A peaks were upregulated and 1,915 peaks were downregulated. It was reported that m6A peaks were mainly concentrated in long exons, stop codons, and in 3′UTRs (Dominissini et al., 2012). Our results also showed that these peaks were mainly concentrated in the 3′UTRs region. Based on the combined analysis of transcriptome and differential peaks, 19 differentially expressed genes containing RNA methylation modification were screened and all of them were related to cashmere growth. The FPKM of these genes in CT-LCG was higher than that in FT-LCG. According to published data, a consistent motif sequence “RRACH” is over-expressed in the m6A motif region. However, in our current data, another motif sequence, GAAGA, was enriched by MEME and HOMER software. The specific reasons are not clear, which need to be further studied. In addition, GO and KEGG pathway analysis were performed to deduce potential functions of m6A modified genes. Some pathways are known to play a vital role in the regulation of hair follicle development, such as Wnt, TGF-β, Notch, and MAPK which have 7, 6, 1, 13 differentially expressed genes, respectively. In this study, the m6A regulatory level of the differentially selected genes was negatively correlated with the transcriptional level in the cashmere goats' skin. Therefore, it can indicate that m6A could regulate mRNA degradation. The expression of differential genes in the skin tissue of CT-LCG were higher than that of FT-LCG in the RNA-seq result. Quantitative real-time PCR (qRT-PCR) confirmed that these differently methylated genes did exist in the skin tissue of cashmere goats.

As an important part of the skin-specific genetic network, EGR3 has been found to be related to the keratinocyte differentiation-related module and contribute to late epidermal differentiation (Kim et al., 2019). Keratin, as a structural protein of ectodermal cells, constitutes the hair skeleton, protects cells from mechanical damage, participates in cell signal transduction and apoptosis, and has functions of influencing hair diameter, participating in hair structural differentiation and skin appendage organ formation (Jin et al., 2011; Liffers et al., 2011). KRT family and KAP family are the main structural proteins of cashmere fiber, which determine the quality of cashmere fiber cluster (Rogers et al., 2004). Studies found that KAP1.1, KAP1.3, KAP8.1, KAP7.1, and KAP8.2 were related to cashmere fiber growth (Rogers et al., 2004; Jin et al., 2011; Plowman et al., 2012), we also identified these genes in our sequencing results. As an acidic type I keratin gene, KRT26 may be involved in the morphogenesis of hair follicles (Zhao et al., 2009). KRT32, and KRT82 mRNA were localized to cells forming the wool fiber and its cuticle in sheep. The study has shown that loss of ZDHHC21 function in hair loss mice results in defects in interfollicular epidermis, sebaceous gland hyperplasia, and delay in hair follicle differentiation (Pleasantine et al., 2009). LOC102176522, LOC108638295, LOC106503204, LOC102173780, LOC108634870, GJA1, GJB6, FRS2, and CTSK are also modified by m6A, but little research has been done on them, which may also be related to cashmere growth.

FZD6 is involved in regulating the Wnt signaling pathway. Previous studies have proved that Wnt signaling pathway plays a key role in maintaining hair follicle induction and forming new hair fibers in human and mouse data (Bai et al., 2017). The Wnt signaling pathway can regulate hair growth cycle and promote hair follicle differentiation (Andl et al., 2002). In addition, CAMK2G, FZD6, KANK3, LEF1, LYG2, MXRA8, and VANGL2 were enriched in the Wnt signaling pathway in our results. The classical Wnt signaling pathway participates in the biological process of skin and has the functions of regulating epithelial morphology, hair follicle development, and related cell differentiation (Andl et al., 2002). LEF1 is a downstream transcription factor of Wnt signaling pathway. Many experiments have shown that when Wnt signaling pathway is activated, LEF1 promotes hair follicle stem cells to differentiate into cells constituting hair follicle structure and plays an important role in hair follicle development (Ito et al., 2007). Other pathways related to cashmere growth, such as Notch, MAPK, and TGF-β signaling pathways, have m6A-modified genes enrichment. The role of most genes has not been reported in hair follicle growth and development. Notch signaling pathway can affect the formation of epidermal appendages. Notch signal is involved in the growth and development of various hair follicle cells. There were 13 differentially expressed genes were enriched in MAPK signaling pathway. It has been reported that the MAPK signaling pathway may be related to hair color formation (Schellenberger et al., 2011). MAPK signaling pathway plays an important role in regulating hair cycle and self-renewal of hair follicle stem cells (Akilli Öztürk et al., 2015). They are all modified by RNA N6-methylation, and they are involved in regulating these signaling pathways. It can be inferred indirectly that m6A may regulate these signaling pathways to some extent, which may regulate hair follicle development and differentiation.

In summary, this study analyzed the modification of m6A methylation in fine and coarse type Liaoning Cashmere Goat skin tissue. It was suggested that m6A methylation may play a role in regulating the expression of genes related to cashmere growth and cashmere fineness. This study suggests that KRT26, KRT32, KRT82, EGR3, and FZD6 are most likely to play a key role in regulating cashmere growth and fineness. In addition, the data obtained by high-throughput sequencing provide a basis for future research on the function of m6A methylation modification in cashmere growth process.

## Data Availability Statement

The raw data has been made publically available. SRA accession: PRJNA591317.

## Ethics Statement

The animal study was reviewed and approved by Shenyang Agricultural University.

## Author Contributions

Data curation: YW. Formal analysis: YW, XJZ and YZ. Funding acquisition: ZW and WB. Methodology: SupG, ZW, and WB. Project administration: WB. Resources: DG and SulG. Software: YW and TH. Supervision: ZW and XHZ. Validation: YW, JS, CY, ZB and WC. Writing–original draft: YW. Writing–review and editing: YW, YF, ZW, and WB.

## Funding

The work was supported financially by grants from the National Natural Science Foundation of China (No. 31802038, 31872325, 31672388), Key Project Foundation of Education Department of Liaoning Province, China (NO. LSNZD201606), Breeding project of new Liaoning cashmere goat “meat and meat dual-use” (No. 2017202005), Science and Technology Innovation Talent Support Foundation for Young and Middle-aged People of Shenyang City, China (RC170447).

## Conflict of Interest

The authors declare that the research was conducted in the absence of any commercial or financial relationships that could be construed as a potential conflict of interest.
