# Supplementary material for: m6A Methylation Analysis of Differentially Expressed Genes in Skin Tissues of Coarse and Fine Type Liaoning Cashmere Goats
Source: Front Genet. 2020 Jan 22;10:1318. doi: 10.3389/fgene.2019.01318 (PMC6987416; doi:10.3389/fgene.2019.01318)
Supplement: Supplementary file 1 [file Table_1.docx]

**Supplementary Table 1** Sequences of primers used for qRT-PCR analysis.

| Name | Sequences |  | Product size(bp) |
| --- | --- | --- | --- |
| LOC108636561 | F :5' TCCATCTGCTCCAGTGACCT 3' | R :5' GAGGTGCAACAGGAAGGCT 3' | 215 |
| LOC102176522 | F :5' GCAAAGGGCTTAGTGGGG 3' | R :5' TTGACGGTGACTTGGTGGAT 3' | 223 |
| LOC102184693 | F: 5' GCCATTCCTACCGCAACA 3' | R: 5' GGGCCACGCATAACTCACT 3' | 215 |
| LOC108638295 | F: 5' ATGGTCAGCTCCTGTTGTGG 3' | R: 5' GCATCTGGGGCGGCA 3' | 87 |
| LOC106503204 | F: 5' ATGGTCAGCTCCTGTTGTGG 3' | R:5' GCAGCAGGTCTCTTGGCAG 3' | 75 |
| LOC102173780 | F: 5' ATAGGCAGCCATATTTGACATC 3' | R:5' AGCGGTAAGGGACAGAAGTG 3' | 105 |
| LOC108634870 | F: 5' CCGGAAGGTTGGGGTCTA 3' | R: 5' TGTGGGCTGATAAGGGTTTT 3' | 81 |
| LOC102184223 | F:5'TTCGTGGTTCTAAAGAAGGATG 3' | R: 5' GTCTGAGATGTGGGCGTTG 3' | 147 |
| KRT79 | F: 5' AATCTGGAGCCCCTCTTCG 3' | R:5'GCTTGTTGATTTCATCCTCATACT 3' | 154 |
| KRT82 | F: 5' GAAGGTGTTGGACCCGTGA 3' | R: 5' CCGCCTGCTCCCATAGAC 3' | 201 |
| KRT32 | F: 5' GCGGCAGAACCAGGAGTA 3' | R: 5' TGGGAGGGAGTGGAGCAT 3' | 132 |
| KRT26 | F: 5' CTGGCACTGGTCGGCTAA 3' | R: 5' CAGGCGGTCATTGAGGTTC 3' | 236 |
| GJA1 | F: 5' ATGACAAATCCTTCCCAATCTC 3' | R: 5' TTCACCTTGCCGTGCTCT 3' | 241 |
| GJB6 | F: 5' GGTCACCGTCCTCTTTGTCT 3' | R: 5' AGTCCTCCTGCTCGTCCC 3' | 80 |
| VANGL2 | F: 5' CCTGCCCTCCTCAACCTG 3' | R: 5' ACTGACCCGTGGAGTTGTTG 3' | 106 |
| ZDHHC21 | F:5' AGACTCCCTGAGAACCCAAAG 3' | R:5'AAAGAAAACATCAGTGCGTAGC 3' | 242 |
| FZD6 | F: 5' TTCAGATACCCAGAGCGACC 3' | R:5'TGCCTAGCAAGAATCCGATAA3' | 88 |
| FRS2 | F: 5' CCTCTGCCTCCGACGCT 3' | R:5' CACGGGTTCCTCCACTACAT 3' | 175 |
| EGR3 | F: 5' GCACTCAGCACGCAGACCT 3' | R: 5' TCGGGAATCATGGGGAAG 3' | 224 |
| GAPDH | F: 5' TGTTTGTGATGGGCGTGAA 3' | R:5'AGTCTTCTGGGTGGCAGTGAT3' | 173 |
